# Comparative Study of Cellulose Extraction from Wheat Straw: Structural, Morphological, and Thermal Characterisation

**DOI:** 10.3390/foods15203702

**Published:** 2026-10-17

**Authors:** Shingissov Azret Utebaevich, Yerkebayeva Saparkul Umirtaevna, Khamitova Barna, Mukhtarova Gaukhar, Turlybek Madina Abdinabikyzy, Alimkhanov Meiirzhan, Norhashila Hashim, Bernard Maringgal

**Affiliations:** 1 Department of Textile and Food Engineering, Faculty of Technology and Safety of Food Products, M. Auezov South Kazakhstan University, Shymkent 160000, Kazakhstan; azret.utebaevich@mail.ru (S.A.U.); erkesapash@mail.ru (Y.S.U.); barno-007@mail.ru (K.B.); gaukhar961@gmail.com (M.G.); madinaturlibek99@gmail.com (T.M.A.); 2 Agricultural Cooperative Melon Juice Co., Shardara, Turkistan 161400, Kazakhstan; alimhanov.meirzhan@gmail.com; 3 Department of Biological and Agricultural Engineering, Faculty of Engineering, Universiti Putra Malaysia, Serdang 43400, Selangor, Malaysia; norhashila@upm.edu.my; 4 SMART Farming Technology Research Centre (SFTRC), Faculty of Engineering, Universiti Putra Malaysia, Serdang 43400, Selangor, Malaysia; 5 Faculty of Resource Science and Technology, Universiti Malaysia Sarawak, Kota Samarahan 94300, Sarawak, Malaysia; 6 UNIMAS Agro Research Center, Universiti Malaysia Sarawak, Kota Samarahan 94300, Sarawak, Malaysia

**Keywords:** acidified sodium chlorite, alkaline hydrogen peroxide, cellulose, peracetic acid extraction, wheat straw

## Abstract

The growing demand for sustainable and biodegradable materials has increased interest in utilising agricultural residues as renewable sources of cellulose. Wheat straw is an abundant lignocellulosic by-product with significant potential for value-added applications. However, the efficiency of different cellulose extraction methods and their effects on cellulose properties remain insufficiently compared. This study evaluated three chemical extraction methods, i.e., acidified sodium chlorite (ASC), alkaline hydrogen peroxide (AHP), and peracetic acid (PAA), for isolating cellulose from wheat straw. The extracted cellulose was characterised in terms of extraction yield, chemical structure, crystallinity, surface morphology, and thermal stability using Fourier-transform infrared spectroscopy (FTIR), X-ray diffraction (XRD), scanning electron microscopy (SEM), and thermogravimetric analysis (TGA). PAA produced the highest cellulose yield (40.91%), followed by AHP (39.33%) and ASC (23.41%). FTIR spectra confirmed the effective removal of lignin and hemicellulose by all three methods while preserving the cellulose structure. XRD analysis showed increased crystallinity after extraction, with PAA exhibiting the highest crystallinity index (74.58%), followed by AHP (72.97%) and ASC (72.09%). SEM observations revealed that PAA generated a more porous and disrupted fibre morphology, whereas ASC better preserved the fibre structure. Thermal analysis indicated that ASC-extracted cellulose exhibited the greatest thermal stability, while PAA and AHP provided higher cellulose recovery and greater fibre opening. Overall, the results demonstrate that the choice of extraction method significantly influences the physicochemical properties of wheat straw cellulose. PAA is the most suitable method when high cellulose yield and crystallinity are desired, whereas ASC is preferable for applications requiring enhanced thermal stability. The extracted cellulose shows considerable potential as a sustainable reinforcement material for biocomposites, biodegradable food packaging, particle boards, and other bio-based products.

## 1. Introduction

Interest in materials from lignocellulosic biomass that are renewable and biodegradable has increased due to concerns over environmental sustainability and the dependence on petroleum-based resources [1]. Cellulose, the most abundant natural polymer, has attracted considerable attention because of its renewability, biodegradability, and favourable mechanical properties [2]. Agricultural residues such as wheat straw are considered a promising feedstock for cellulose extraction because of their relatively high cellulose content and wide availability [3]. Wheat straw is a by-product of the cultivation of wheat, one of the most widely grown cereal crops in the world, with production of 790 million tons in 2023/2024 [4]. Large amounts of wheat straw are generated each year, particularly in agricultural regions, but much of it is not fully utilised and is often disposed of by open burning [5]. This practice releases greenhouse gases and other pollutants while also resulting in the loss of a potentially valuable biomass resource [6]. Wheat straw is mainly composed of cellulose, hemicellulose, and lignin, making it a suitable raw material to produce value-added products [7]. However, the use of wheat straw for cellulose production is still relatively limited despite its availability as a renewable lignocellulosic resource [8]. A key challenge in cellulose extraction is the removal of lignin and hemicellulose while maintaining the structure and properties of cellulose [5]. Cellulose obtained from wheat straw can be used in a range of applications, including biodegradable packaging, paper and pulp products, biocomposites, pharmaceuticals, and polymer reinforcement materials [2]. Therefore, effective isolation methods are needed to improve the utilisation of agricultural residues and support the development of a circular bio economy [3].

The isolation of cellulose from wheat straw generally involves the removal of lignin and hemicellulose through chemical extractions [6]. One of the commonly used methods is acidified sodium chlorite (ASC) extraction, which is effective for lignin removal and can produce cellulose with relatively high purity [9]. Alkaline hydrogen peroxide (AHP) extraction is considered a relatively environmentally friendly method. It combines alkaline treatment with peroxide bleaching to remove lignin and hemicellulose under milder conditions than some conventional extraction methods [6,10]. Previously, isolation of cellulose from wheat straw using alkaline hydrogen peroxide successfully obtained 79% cellulose using this method [7]. Peracetic acid (PAA) has recently gained attention as a green oxidising agent for biomass pre-treatment. It is biodegradable, relatively low in toxicity, and effective in removing lignin while maintaining the cellulose structure [11]. PAA has also been used for cellulose extraction from maize husk, with a reported yield of 75.1% [9]. Furthermore, PAA has also been used for cellulose extraction from different biomass sources. For example, cocoa pod husk produced a cellulose yield of 69.39% [11], while coffee hulls gave 71.61% cellulose with increased crystallinity [12]. Overall, this method is suitable for cellulose isolation and can produce material that is useful for applications such as nanocellulose and biocomposites [7,13].

Numerous studies have explored different chemical methods for cellulose isolation, including ASC extraction and AHP extraction [7,9]. While both methods have demonstrated effectiveness in extracting cellulose, they present several limitations. ASC extraction, although capable of achieving high cellulose purity and yield, involves the use of strong oxidising agents and acidic conditions, which may raise environmental and safety concerns [6,14]. In contrast, AHP extraction is considered more environmentally friendly; however, it may require optimised conditions to achieve comparable efficiency in lignin removal and cellulose purity [15]. Emerging green methods such as PAA extraction have also shown promising results due to their biodegradability and ability to selectively remove lignin [11,15]. However, studies comparing PAA with conventional extraction methods are still limited. Most available work focuses on comparisons between only two methods [7], so the relative performance of multiple extraction techniques under similar conditions is not yet well established.

The properties of isolated cellulose depend on the extraction method used; hence, proper characterisation is needed to assess how well each technique works [13]. Fourier-transform infrared spectroscopy (FTIR) is often used to check the removal of lignin and hemicellulose by looking at changes in characteristic functional groups [6]. X-ray diffraction (XRD) is used to examine the crystallinity and structural arrangement of cellulose [5]. Scanning electron microscopy (SEM) is used to observe surface morphology and fibre characteristics, while thermogravimetric analysis (TGA) assesses thermal stability and decomposition behaviour [12]. Together, these techniques give a general understanding of the physicochemical properties of cellulose [7]. Another gap is in how cellulose from different extraction methods is fully characterised [16]. FTIR, XRD, SEM, and TGA are often used, but these results are not always discussed together, so the link between the extraction method and cellulose properties is still not very clear [5]. Differences in crystallinity, thermal stability, and morphology can influence how suitable cellulose is for different applications [13,17], but these relationships are not always compared in a systematic way. In addition, the effects of different chemical extraction methods on the structure and properties of wheat straw cellulose are still not fully clear. Variations in oxidation strength, reaction conditions, and lignin removal efficiency can lead to differences in cellulose quality, which then affects its potential applications [3].

Thus, a comparative study of multiple extraction methods, including ASC, AHP, and PAA, is needed. Such a study should combine different characterisation techniques to better understand how each method affects the structure, morphology, crystallinity, and thermal stability of the extracted cellulose. This work aims to address these gaps by comparing three isolation methods in detail, contributing to more efficient and sustainable approaches for cellulose extraction from wheat straw.

## 2. Materials and Methods

### 2.1. Wheat Straw Powder Preparation

The wheat straw powder (WSP) used in this study originated from Shymkent, Kazakhstan and is a by-product of the wheat (*Triticum aestivum*) harvest. The WSP was dewaxed by Soxhlet extraction (BÜCHI Labortechnik AG, Flawil, Switzerland) using ethanol (Bendosen; Progressive Scientific Sdn. Bhd., Batu Caves, Selangor, Malaysia) at an ethanol-to-WSP ratio of 1:20 (*w*/*v*) for 3 h. The dewaxed wheat straw powder (DWS) was subsequently dried prior to cellulose extraction [7].

### 2.2. Cellulose Isolation Method

#### 2.2.1. Acidified Sodium Chlorite Extraction

The sodium chlorite extraction procedure was conducted following the method outlined by a previous study, with some modification [7]. A sample of about 2.5 g was suspended in 80 mL of heated water at 80 °C. The suspension was then bleached by the addition of a solution consisting of 0.5 mL of acetic acid (Sigma-Aldrich, St. Louis, MO, USA) and 1 g of sodium chlorite (Sigma-Aldrich, St. Louis, MO, USA), and subjected to heating in a water bath at 80 °C for 4 h. The procedure was conducted four times. Subsequently to the treatment, the remaining white sample (holocellulose) was subjected to sieving and rinsed with sufficient distilled water, followed by drying in an oven at 75 °C for 24 h. After the procedure, the holocellulose underwent treatment with a 17.5% sodium hydroxide (NaOH) (Sigma-Aldrich, St. Louis, MO, USA) solution (100 mL) at ambient temperature for 30 min. The process was performed twice. The residue was filtered and then washed with distilled water to achieve a pH of 7. The residue (cellulose) was then dried in an oven at 75 °C prior to further application.

#### 2.2.2. Alkaline Hydrogen Peroxide Extraction

The alkaline hydrogen peroxide extraction was conducted following the method outlined by a previous study, with some modification [7]. The DWS was bleached using a preheated mixture of hydrogen peroxide (20% *v*/*v*; Sigma-Aldrich, St. Louis, MO, USA) and NaOH (1% *w*/*v*) at a solid-to-liquid ratio of 1:20 (g/mL) to remove the remaining lignin and hemicellulose under constant magnetic stirring. The selected liquid-to-solid ratio provided sufficient dispersion of the biomass particles and facilitated the generation of reactive peroxide species for oxidative delignification. The bleaching treatment was performed at a temperature of 65 °C for 90 min [10]. The cellulose-rich residue was then filtered and washed with distilled water until neutrality. The last phase was drying the cellulose at 75 °C prior to further application.

#### 2.2.3. Peracetic Acid Extraction

The PAA extraction procedure was conducted following the method outlined by a previous study with some modification [4]. PAA extractions of wheat straw were conducted in a 250 mL Erlenmeyer flask connected to a water-cooled condenser. A PAA solution was prepared by mixing acetic acid and hydrogen peroxide (1:1, *V*/*V*). The concentration of acetic acid was 2.0 M in the reaction. The maximum concentration of PAA can be estimated to be 2.0 M if the reaction between acetic acid and hydrogen peroxide was complete. For each batch, dry, milled wheat straw (5 g) was suspended in 100 mL of distilled water before the addition of the PAA solution (25 mL). A varying amount of sulfuric acid (200 mM; Sigma-Aldrich, St. Louis, MO, USA) was added directly to the reaction vessel, and the reaction was allowed to proceed at 75 °C for 22 h under magnetic stirring. The reaction was stopped by cooling down to room temperature, and the mixture was filtered. The resulting solid fraction was washed once with 100 mL of 0.01 M sodium hydroxide solution and filtered, then washed repeatedly with distilled water, and filtered again until pH neutral.

### 2.3. Physicochemical Characterisation of Extracted Cellulose

#### 2.3.1. FTIR Analysis

The solid components were dried at 60 °C for 24 h before FTIR measurement. The Spectrum 100 FTIR spectrometer ( PerkinElmer, Shelton, CT, USA) was loaded with 1 g of solid at 4 cm^−1^. The 4000 to 400 cm^−1^ spectral range was chosen [18]. The purity of extracted cellulose was not directly quantified in this study. Instead, the removal of major non-cellulosic components, particularly lignin and hemicellulose, was assessed through FTIR analysis, while XRD, SEM, and TGA were used to evaluate the structural, morphological, and thermal characteristics of the extracted cellulose.

#### 2.3.2. XRD Analysis

The cellulose fraction’s crystallinity index was measured using an XRD-6000 (Shimadzu Corporation, Kyoto, Japan). The diffraction angle (2θ) was scanned from 0° to 45° at 1° per minute with a 0.05° step size [19]. The crystallinity index was calculated using the amorphous portion’s intensity values and crystalline fraction’s highest intensity (*I*_002_) according to Equation (1).

(1) $$\mathrm{Crystalline}\;\mathrm{Index},\;\mathrm{CrI}\;(\%)=\frac{\left(I_{002}-I_{\mathrm{am}}\right)}{I_{002}}\times 100$$

#### 2.3.3. SEM Analysis

A SEM model S-3400N (Hitachi High-Tech Corporation, Tokyo, Japan) was used to analyse the solid fraction morphology in wheat straw cellulose. Morphological SEM pictures were taken at 2000× and 6000×. Each sample was gold-coated for 120 s using an EM scope SC500 Sputter Coater (Quorum Technologies, Laughton, UK) [19]. The photos were taken in a vacuum with 10 kV accelerating voltage. A 4500× magnification was used, with a working distance of 15 to 16 mm.

#### 2.3.4. TGA Analysis

For TGA analysis, about 0.5 g was placed in an aluminium pan on the platinum basket of the furnace in a thermogravimetric analyzer by Mettler-Toledo GmbH (TGA/DTG 1, Columbus, OH, USA) and heated from 10 °C to 600 °C at a heating rate of 5 °C/min using nitrogen as carrier gas at a flow rate of 100 mL/min [20]. The weight losses of the cellulose samples were monitored to determine their differential thermal profiles; the TGA and derivative thermogravimetric (DTG) graphs were generated from the analysis.

## 3. Results and Discussion

### 3.1. Cellulose Extraction Yield

**Figure 1:**
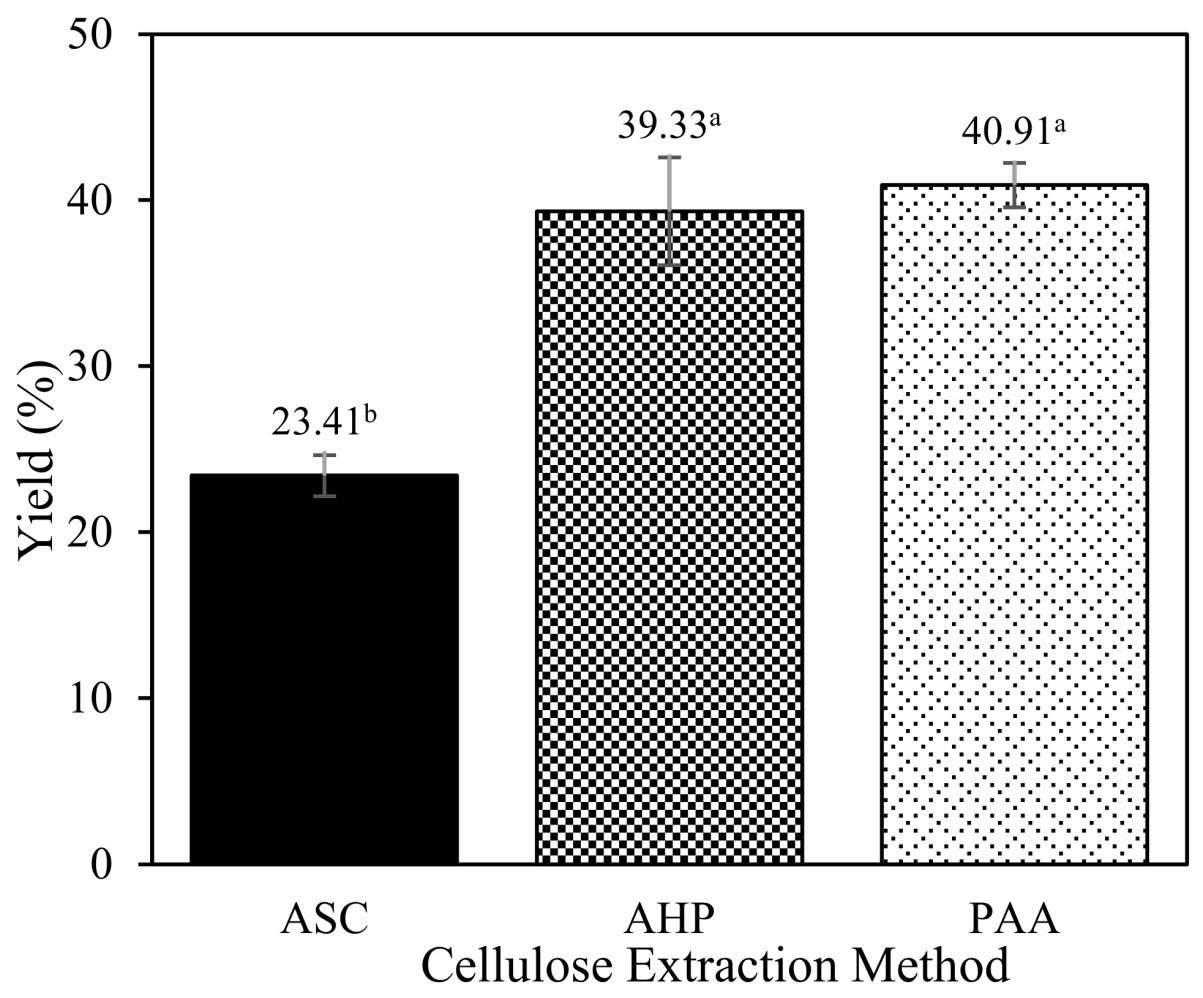
Cellulose extraction yield obtained using acidified sodium chlorite (ASC), alkaline hydrogen peroxide (AHP), and peracetic acid (PAA) extraction methods. Data represent the mean of three replicates. Different letters indicate significant differences at *p* < 0.05 according to Tukey’s test.

The three extraction methods studied showed significant differences (*p* < 0.05) in the yield of cellulose obtained from WSP. The lowest recovery was obtained using ASC (23.41%), followed by AHP (39.33%) and PAA (40.91%), as shown in Figure 1. These observed variations can be attributed to the differing selectivities of each technique toward lignin, hemicellulose, and cellulose. Although the initial chemical composition of wheat straw was not experimentally determined in this study, the extraction yield obtained here represents the recovered cellulose fraction relative to the initial dry biomass weight. Therefore, differences in yield should be interpreted as the combined effects of lignin and hemicellulose removal, cellulose preservation, and possible losses of soluble carbohydrate fractions during extraction. Previous studies have shown that wheat straw composition can vary depending on cultivar, cultivation conditions, and processing history, which influences the theoretical cellulose recovery achievable during fractionation. Ultimately, the final yield reflects the balance between removal of non-cellulosic components, preservation of cellulose structures, and possible losses of soluble fractions during the extraction process [10].

Among the extraction methods, ASC produced the lowest yield at 23.41%. This method generates chlorine dioxide, a powerful oxidising agent that effectively breaks down and dissolves lignin [21]. This promotes effective delignification and removal of non-cellulosic components; however, the aggressive oxidative environment may also increase dissolution of soluble fractions, resulting in lower recovered solid yield. Therefore, the lower ASC yield should be interpreted as greater removal of biomass components during fractionation rather than direct evidence of cellulose degradation. Since the initial cellulose content of the raw wheat straw was not quantified, the present study does not calculate theoretical cellulose recovery. Therefore, the ASC yield should be interpreted as a recovered cellulose-rich fraction based on initial biomass weight rather than absolute cellulose recovery efficiency. This is aligned with previous studies showing that the ASC method is highly effective for lignin removal [7]. Conversely, AHP resulted in significantly higher yield at 39.33%. Hydrogen peroxide is more effective on lignin and less destructive on the carbohydrate fraction in alkaline conditions [6]. Furthermore, the treatment promotes fibre swelling, which enhances delignification without causing extensive cellulose degradation [19]. The high cellulose recovery obtained via AHP (39.33%) was facilitated by the adequate liquid-to-solid ratio (1:20 g/mL), which ensured sufficient dispersion of reactive peroxide species, particularly hydroperoxyl ions (HOO^−^), and effective swelling of the biomass matrix without causing severe carbohydrate dissolution. The alkaline peroxide system promotes the generation of active oxygen species that contribute to lignin oxidation while maintaining cellulose integrity under controlled reaction conditions. Consequently, more solid material was retained post-extraction, explaining the higher recovery for AHP. These observations agree with previous findings, where AHP was shown to achieve effective delignification while preserving a significant proportion of polysaccharides [9,16].

In contrast, PAA produced the highest yield among the three methods, reaching 40.91%. This result is likely attributable to its higher selectivity for lignin and relatively mild effect on cellulose [22]. PAA effectively oxidises and solubilises lignin while preserving most of the carbohydrate structure, thereby minimising material losses during processing [5]. Comparisons of oxidative delignification methods demonstrate that PAA is generally more selective than ASC and less damaging to the cellulose structure [23]. These findings align with previous research, where PAA achieved higher yield as compared to ASC and AHP in extracting celluse from corn husk [9]. In addition, the elevated yields obtained via AHP and PAA reflect superior preservation of the carbohydrate fraction, whereas the lower yield from ASC indicates a more aggressive removal of biomass components during extraction.

### 3.2. Functional Group Changes

**Figure 2:**
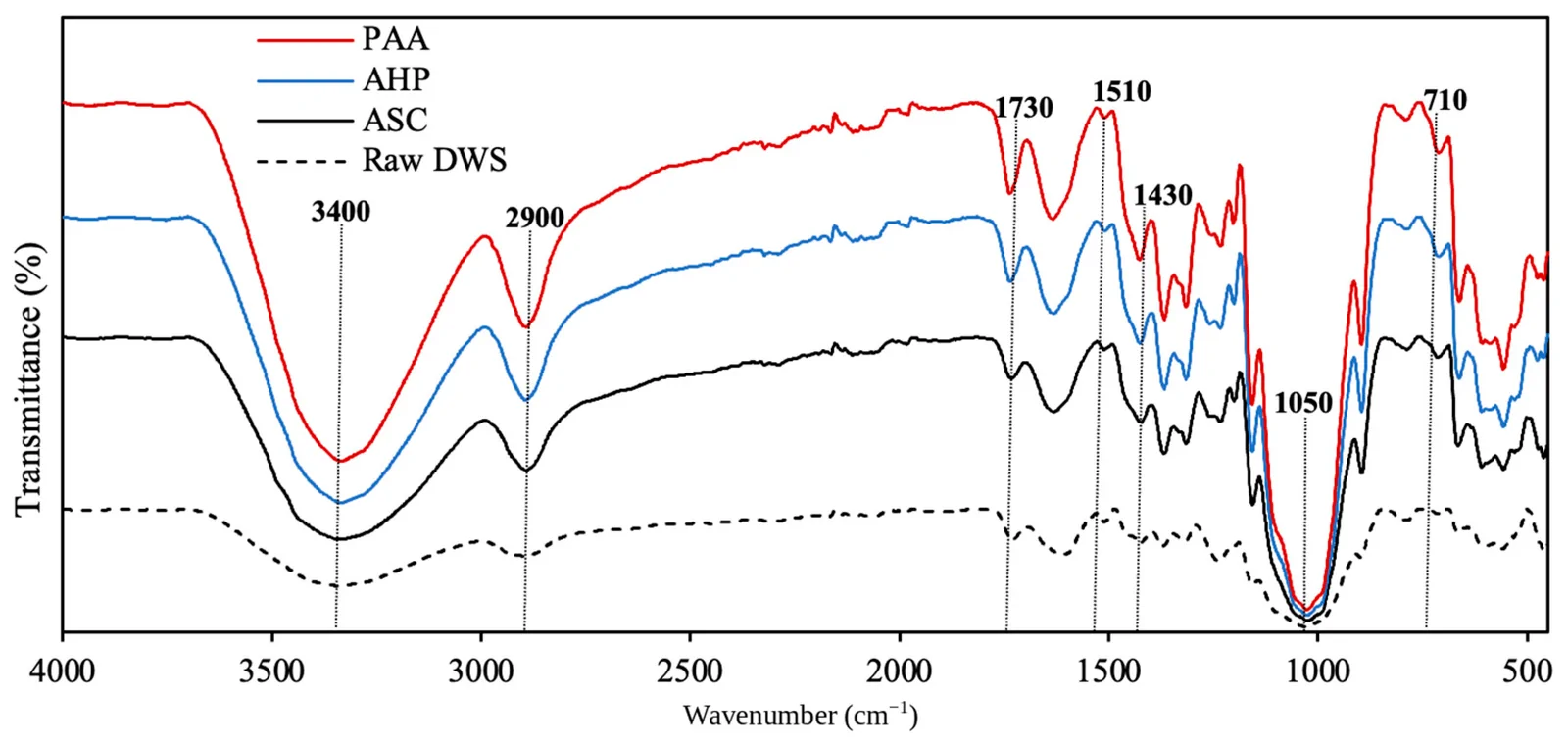
Fourier-transform infrared spectroscopy (FTIR) spectra of raw dewaxed wheat straw (DWS) and cellulose extracted using acidified sodium chlorite (ASC), alkaline hydrogen peroxide (AHP), and peracetic acid (PAA).

The FTIR spectra of raw DWS and cellulose extracted using the different methods are shown in the Figure 2. The similarity of the FTIR spectra suggests that the cellulose fraction was retained during the chemical extraction method in all the samples. The broad absorption band of raw DWS at 3400 cm^−1^, corresponding to hydroxyl (–OH) group stretching vibrations, shifted to narrow peaks in the PAA, ASC, and AHP samples, indicating the removal of the hemicellulose and lignin. This delignification exposed a greater proportion of cellulose, as evidenced by the prominent peak at 2900 cm^−1^ attributed to aliphatic hydrocarbon (C–H) bond stretching. A similar observation was reported by Amensisa et al. [16] and Chand et al. [6] during AHP extraction of cellulose from coconut husk and coffee husk, where comparable peaks at 2900 cm^−1^ and 3400 cm^−1^ signalled enhanced cellulose exposure following lignocellulosic matrix breakdown. Furthermore, the intensity of the peaks at 1730 cm^−1^ (C–O stretching vibration in hemicellulose) and 1510 cm^−1^ (lignin aromatic C–O vibration) decreased substantially in extracted samples. Comparable reductions were noted in PAA-extracted cellulose from WSP by Rashidi et al. [18], as well as in AHP and ASC extracted WSP cellulose by Qasim et al. [7]. Additionally, Vu et al. [19] confirm that the complete absence or attenuation of the 1510 cm^−1^ peak reflects extensive delignification during AHP treatment, a trend likewise observed in PAA-extracted corn husk cellulose [23]. Overall, the significant reduction or disappearance of these non-cellulosic absorption band confirms the successful removal of lignin and hemicellulose, yielding a highly purified cellulose material.

Meanwhile, the absorption bands at 1430 cm^−1^ and 1050 cm^−1^ became markedly more pronounced following ASC, PAA, and AHP extractions. The peak at 1430 cm^−1^ is assigned to CH_2_ bending vibrations and has been widely associated with cellulose crystalline ordering due to its sensitivity toward changes in cellulose chain packing and lattice structure [23,24]. Nelson and O’Connor reported that infrared bands in the 1400 cm^−1^ region can be used to monitor changes related to cellulose crystallinity and crystal lattice type. In contrast, the 1050 cm^−1^ band corresponds to C–O and C–O–C stretching vibrations within the cellulose backbone, reflecting the polysaccharide structure of cellulose [24]. The increased intensity and resolution of these peaks suggest that the cellulose fraction became more exposed following the removal of non-cellulosic constituents such as lignin and hemicellulose. Similar observations have been reported by Brahma and Ray [13], who found that the 1430 cm^−1^ band became more evident as non-cellulosic components were removed. The increase in these cellulose-related peaks therefore supports the effectiveness of the extraction treatments in enriching the cellulose fraction.

In addition, the peak at 710 cm^−1^ became more noticeable after ASC, PAA, and AHP extraction. This peak is associated with the cellulose Iβ structure and is commonly observed in crystalline cellulose. Its stronger appearance suggests that cellulose became more evident after the removal of lignin and hemicellulose. Similar observations were reported by Vu et al. [19] and Moghaddam et al. [25], who identified the band around 710 cm^−1^ as a characteristic feature of cellulose Iβ from PAA- and AHP-cellulose-isolated samples. Overall, the FTIR results show that PAA, ASC, and AHP were effective in removing lignin and hemicellulose while retaining the cellulose fraction. This is supported by the reduction of peaks associated with non-cellulosic components and the clearer appearance of cellulose-related peaks after extraction. The more noticeable peaks at 1430, 1050, and 710 cm^−1^ also suggest changes in the cellulose structure and a possible increase in its crystalline regions. However, FTIR alone is not sufficient to determine the degree of crystallinity. Therefore, XRD analysis will be used in the next section to further examine the crystalline structure and calculate the crystallinity index (CrI) of the extracted cellulose.

### 3.3. Cellulose Crystallinity

**Figure 3:**
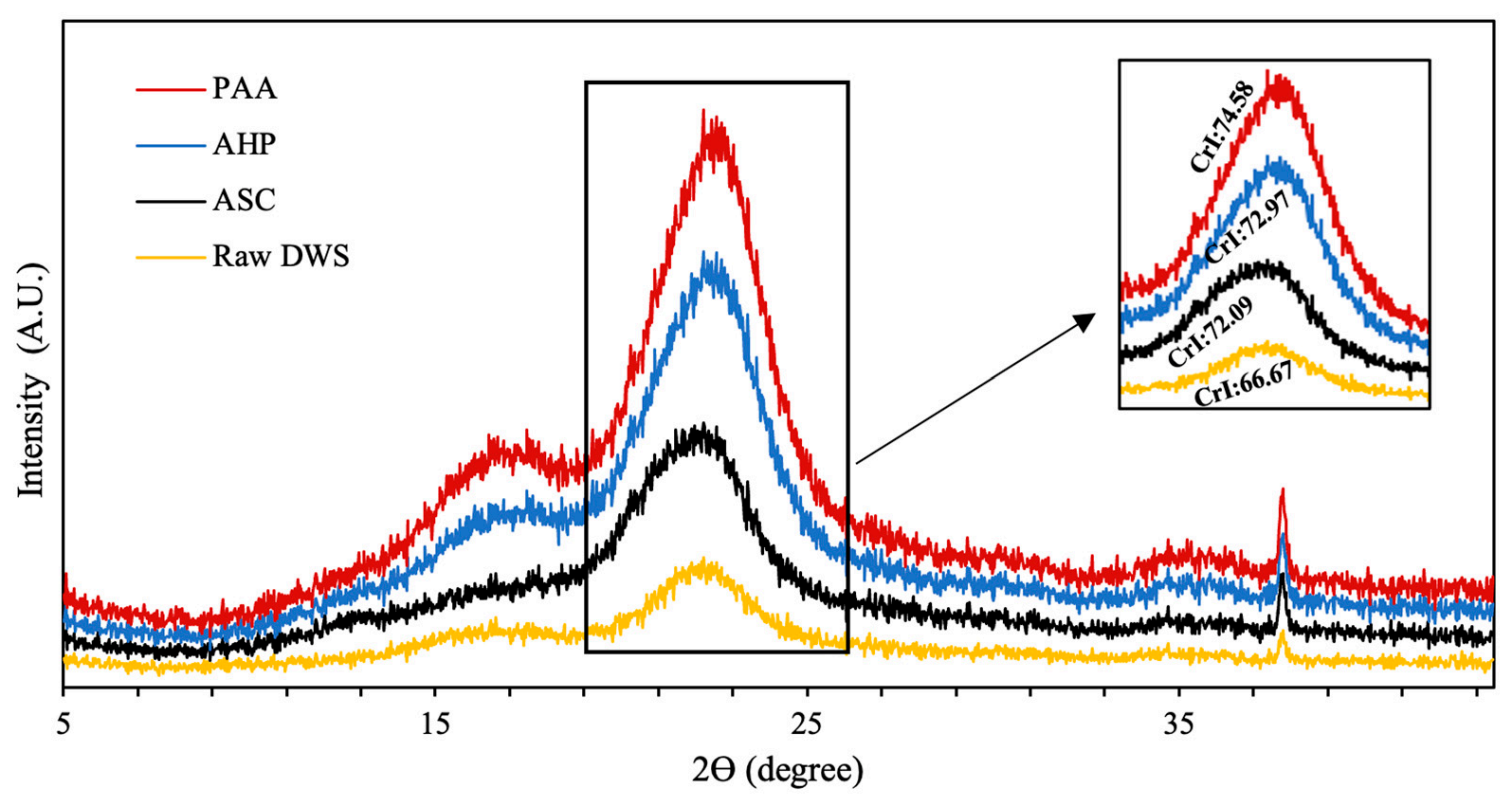
X-ray diffractograms of raw dewaxed wheat straw (DWS) and cellulose extracted using acidified sodium chlorite (ASC), alkaline hydrogen peroxide (AHP), and peracetic acid (PAA), including the calculated crystallinity index (CrI) of the extracted cellulose.

Following different extraction procedures, the crystallinity of the wheat straw cellulose fibre was assessed by X-ray diffraction (XRD) analysis. As shown in Figure 3, raw DWS fibres exhibit a composite of crystalline and amorphous structure. This accounts for the low peak intensity, as the cellulosic core remains encased within the amorphous hemicellulose and lignin matrix. Compared to raw DWS, the cellulose recovered via PAA, AHP, and ASC treatments displayed a markedly higher peak intensity in the *I*_002_ region, confirming the retention and enhancement of crystalline framework. This enhancement implies that effective hemicellulose depolymerisation and delignification were accomplished through chemical bleaching [25,26]. Acidic media selectively degrade the amorphous region while leaving the crystalline domains intact [19]. Among all tested extraction methods (ASC, AHP, and PAA), the PAA extraction method yielded the highest cellulose recovery (40.91%) alongside the highest crystallinity index of 74.58% CrI (Figure 1 and Figure 3). AHP was the next-most-effective technique, producing 39.33% of cellulose with CrI of 72.97%, followed by ASC at 72.09% CrI. The relatively high crystallinity index values obtained in this study are comparable with chemically treated wheat straw cellulose reported in previous studies. Previous studies have reported that bleaching and delignification treatments can increase wheat straw cellulose crystallinity values into the range of approximately 65–75%, depending on the extraction severity and the method used for CrI calculation. Although native wheat straw generally exhibits lower crystallinity due to the presence of amorphous lignin and hemicellulose matrices, chemical extraction processes can increase apparent crystallinity by selectively removing amorphous lignin and hemicellulose fractions and reducing the contribution of amorphous cellulose domains, thereby exposing ordered cellulose regions and increasing the relative proportion of crystalline domains. The removal of amorphous lignin and hemicellulose exposes the ordered cellulose domains, thereby increasing the relative proportion of crystalline regions. Similar increases in cellulose crystallinity following delignification and bleaching treatments have been reported for wheat straw and other lignocellulosic materials [22,27,28].

As illustrated in Figure 3, the diffractograms of the extracted cellulose revealed a sharp, high intensity crystalline peak (*I*_002_) alongside an amorphous trough (*I*_am_) corresponding to the crystallographic plane (*I*_101_), aligning with findings by Jayalaksamana et al. [26]. The prominent (*I*_002_) reflection in PAA-extracted cellulose indicates a superior proportion of ordered crystalline domains relative to ASC and AHP treatments. This observation is consistent with previous reports indicating that PAA treatment effectively promotes lignin removal and increases the apparent crystallinity of extracted cellulose through selective delignification [27,29]. The higher CrI observed for PAA-extracted cellulose can be attributed to the effective oxidative removal of lignin and hemicellulose fractions associated with amorphous regions, together with disruption of lignin–carbohydrate complexes and exposure of previously embedded cellulose domains. These changes increase the relative contribution of ordered crystalline cellulose regions. Similar increases in cellulose crystallinity following peracetic acid-assisted delignification have been reported in lignocellulosic biomass systems. The oxidative activity of PAA toward lignin-related structures facilitates efficient delignification while maintaining the major crystalline cellulose framework observed by XRD analysis. It should also be noted that the CrI values calculated in this study were obtained using the empirical Segal peak-height method. Although this approach is widely used for comparative evaluation of cellulose crystallinity, it may provide higher apparent crystallinity values compared with advanced approaches such as XRD peak deconvolution or spectroscopic methods because the amorphous contribution is estimated from a single diffraction intensity value. Alternative approaches, including FT–Raman spectroscopy, have been investigated for determining cellulose I crystallinity and comparing crystallinity estimates obtained using X-ray diffraction methods [30]. Therefore, the CrI values reported here should be interpreted comparatively among the extraction treatments rather than as absolute crystallinity measurements. The increased CrI indicates a greater proportion of ordered cellulose regions, which may be beneficial for applications requiring enhanced structural rigidity and reinforcement potential.

### 3.4. Morphological Changes

**Figure 4:**
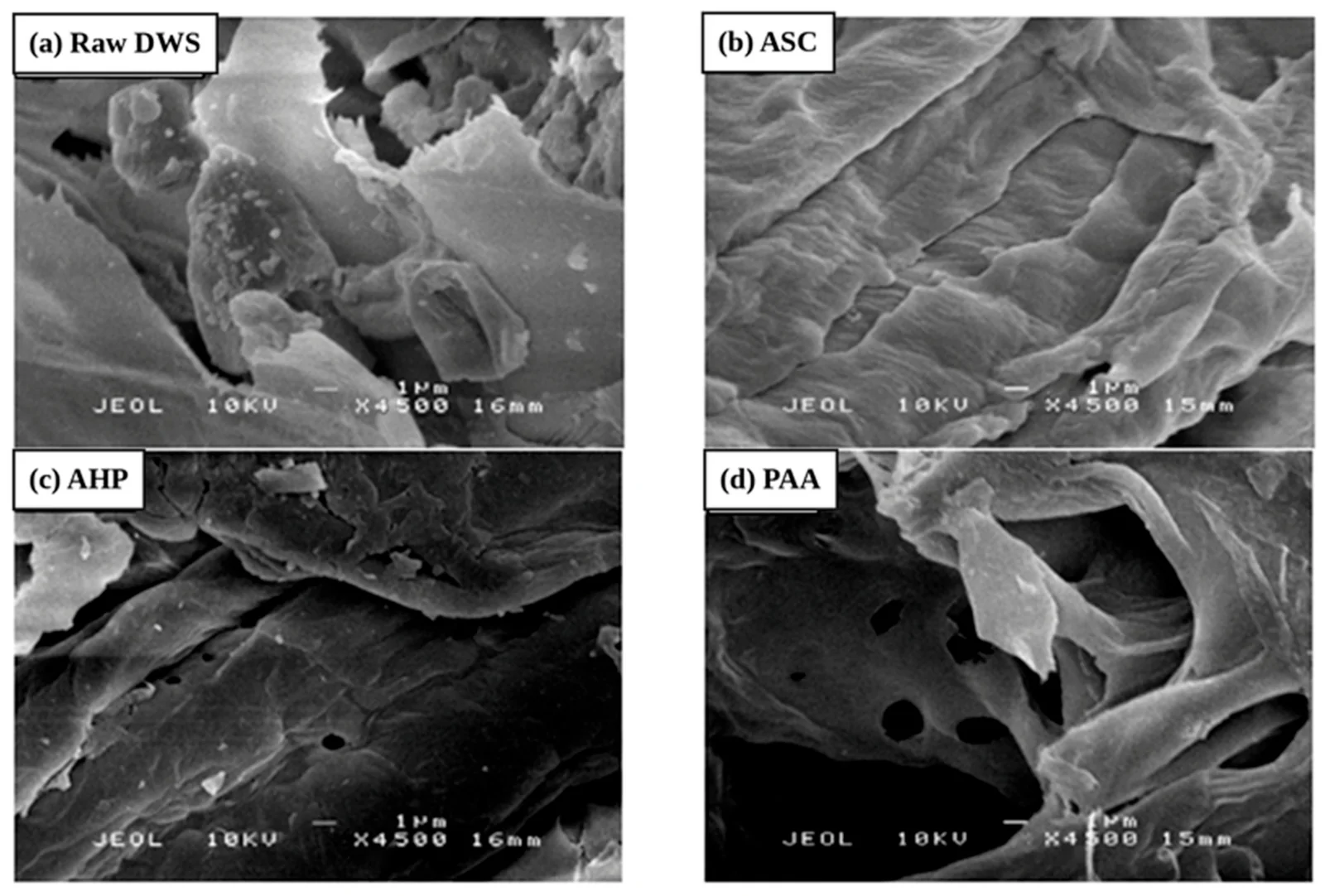
Scanning electron microscopy (SEM) images of (**a**) raw dewaxed wheat straw (DWS) and cellulose extracted using (**b**) acidified sodium chlorite (ASC), (**c**) alkaline hydrogen peroxide (AHP), and (**d**) peracetic acid (PAA).

To better understand the structural transformation of the extracted cellulose, SEM analysis was performed for all samples. As shown in Figure 4a, raw DWS exhibited a relatively smooth, compact surface with no distinct cellulose fibre structure visible, indicating that the cellulosic framework remained embedded within non-cellulosic constituents. Following chemical extraction, the isolated cellulose displayed marked morphological differences compared to the raw precursor (Figure 4). Specifically, cellulose extracted using ASC retained its bundled fibre arrangement, with individual fibrils remaining closely packed and aggregated rather than fully separated (Figure 4b). This morphology suggests that ASC treatment effectively degraded the aromatic lignin matrix while preserving the primary cellulose carbohydrate chains. Consequently, the removal of the surrounding matrix exposes the underlying cellulose fibres without causing significant destruction to their crystalline framework [21,28]. Similar SEM morphological features have been reported for ASC-extracted wheat straw cellulose by Qasim et al. [7].

In contrast, the surface morphology following AHP extraction (Figure 4c) reveals that the individual fibre structure remains visible, though they appear less distinct and less rough compared to ASC-extracted cellulose. According to Amensisa et al. [16], the partial removal of hemicellulose and lignin during AHP extraction resulted in a smoother cellulose surface. Similarly, Chand et al. [6] observed that AHP extraction removes non-cellulosic components while promoting pore formation and increased fibre exposure. The authors further emphasised that the surface morphology of extracted cellulose becomes cleaner and porous after removal of non-cellulosic materials by AHP. Compared with ASC-extracted cellulose, which exhibited cleaner and more separated fibres, AHP-extracted cellulose showed partial fibre bundles and pore formation.

Meanwhile, PAA-extracted cellulose exhibited a rough and irregular surface alongside the formation of noticeable large pores (Figure 4d). The pore formation in PAA cellulose was relatively larger at the same magnification as compared with AHP cellulose. The significant wheat straw structural disruption and larger pore size of the extracted cellulose indicate the efficiency of the PAA extraction and support its role in effective delignification [4]. The PAA extraction oxidised lignin and disrupted the lignin–carbohydrate complex, resulting in fibre disaggregation and the formation of pores in the cellulose structure [12]. Similar morphological features have been reported for PAA-extracted cellulose from corn husk [9] and cocoa pod husk [11]. The raw WSP showed a compact and smooth surface, with the cellulose structure covered by non-cellulosic components. After extraction, the cellulose fibres became more visible in all samples. Differences in surface morphology were observed among the extraction methods, with the PAA-treated cellulose showing greater fibre disruption and larger pore openings. The ASC exhibits the least structural disturbance but reveals cellulose fibrils, whereas the AHP has substantial disruption with the creation of visible pores.

### 3.5. Thermal Stability

**Figure 5:**
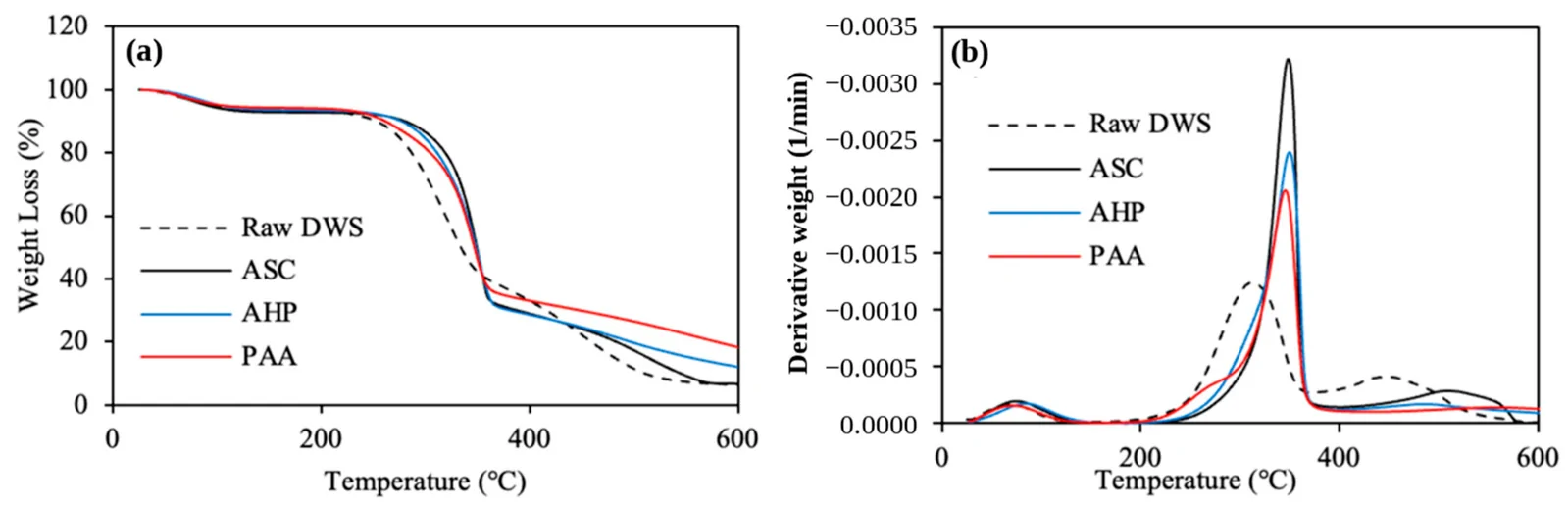
(**a**) Thermogravimetric analysis (TGA) and (**b**) derivative thermogravimetric (DTG) curves of cellulose extracted using acidified sodium chlorite (ASC), alkaline hydrogen peroxide (AHP), and peracetic acid (PAA).

The results of TGA and DTG provide valuable insights into the thermal stability of cellulose extracted from DWS using the ASC, PAA, and AHP methods. The thermal stability of cellulose is crucial for various applications, including biocomposites and bioplastics [5]. The TGA curves of raw DWS, ASC, PAA, and AHP exhibited the main stages of thermal degradation, corresponding to the degradation of hemicelluloses, cellulose, and lignin (Figure 5)**.** Degradation of hemicelluloses occurs in the first stage, which occurs between 200 and 300 °C. The second step involves thermal decomposition of cellulose, which takes place between 300 and 400 °C [7,27]. Degradation was most pronounced in the region around 250 to 330 °C, as seen in Figure 5a TGA curves for PAA, ASC, and AHP-extracted cellulose. However, the higher weight loss of extracted cellulose (PAA, ASC, and AHP) relative to the raw DWS indicates successful cellulose extraction.

Moghaddam et al. [25] observed that cellulose extracted using ASC and AHP experienced higher weight loss at higher temperatures. These modified thermal profiles occurred due to the structural deconstruction and matrix removal [18]. Originally, the cellulose structure is protected by a cross-linked lignin and hemicellulose network. Thus, effective removal and solubilisation of lignin and hemicellulose during PAA, ASC, and AHP treatments resulted in cellulose isolation. Consequently, the removal of these barrier components facilitated weight loss during the main thermal degradation stage.

However, the TGA curves of extracted cellulose (PAA, AHP, and ASC) show higher weight loss between 350 °C and 600 °C compared to raw DWS, with PAA showing the highest weight loss. The removal of thermally resistant lignin is likely the reason for this observation [4]. The raw DWS contains a high percentage of lignin, which does not degrade easily. However, during the extraction process, both lignin and hemicellulose are removed, exposing the crystalline cellulose [22]. Meanwhile, although AHP and ASC also promote lignin and hemicellulose removal, PAA exhibits stronger oxidative activity toward lignin structures, including aromatic linkages and lignin–carbohydrate complexes. This extensive delignification improves cellulose exposure and accessibility; however, the resulting fibre disruption and increased surface area may influence the thermal degradation behaviour of the extracted cellulose [7,31]. Meanwhile, the DTG curve (Figure 5b) shows that the peak of raw DWS is lower and broader. It can be observed that the cellulose extraction method affected the thermal stabilities of extracted cellulose samples as the peaks of ASC, PAA, and AHP increased, became narrower, and shifted to higher temperatures. It is evident that the ASC-extracted cellulose was the most thermally stable, as presented in the previous research [7,32].

This finding can be attributed to the preservation of a more compact fibre structure and higher structural integrity in ASC-extracted cellulose, as observed from SEM analysis. The bundled morphology and reduced fibre disruption may limit heat transfer and delay thermal decomposition, resulting in higher thermal stability. In contrast, although PAA-extracted cellulose exhibited the highest crystallinity index and yield, it showed lower thermal stability compared with ASC. This behaviour does not indicate destruction of the cellulose crystalline structure; rather, it is associated with differences in fibre morphology and surface accessibility. Although crystallinity contributes to structural order, thermal stability is also influenced by fibre morphology, polymer accessibility, residual components, and the degree of structural disruption caused by extraction conditions. The oxidative action of PAA promotes extensive delignification and disruption of lignin–carbohydrate complexes, resulting in a more open porous structure with increased exposed surface area. As observed in SEM analysis (Figure 4d), this morphology provides greater exposed fibre surfaces and accessibility of cellulose chains, which may facilitate earlier thermal decomposition and contribute to the observed weight loss behaviour during TGA analysis.

## 4. Conclusions

Overall, the comparative assessment indicates that AHP and PAA are attractive routes when higher cellulose yield and more open, porous fibre structures are desired, which is beneficial for applications requiring high surface area and strong interfacial bonding, such as biocomposites and reinforced bioplastic. Meanwhile, ASC is more suitable when enhanced thermal stability and relatively preserved fibre structure are prioritised, for example, in applications where dimensional stability at high temperatures is important. The cellulose obtained from all three methods shows properties suitable for incorporation into products such as biocomposites, particle boards, furniture components, and biodegradable food packaging, supporting the valorisation of wheat straw within a circular bioeconomy framework.

## Data Availability

The original contributions presented in the study are included in the article. Further inquiries can be directed to the corresponding author.
